# Socio-Demographic Predictors and Distribution of Pulmonary Tuberculosis (TB) in Xinjiang, China: A Spatial Analysis

**DOI:** 10.1371/journal.pone.0144010

**Published:** 2015-12-07

**Authors:** Atikaimu Wubuli, Feng Xue, Daobin Jiang, Xuemei Yao, Halmurat Upur, Qimanguli Wushouer

**Affiliations:** 1 Department of Epidemiology and Biostatistics, School of Public Health, Xinjiang Medical University, Urumqi, Xinjiang, China; 2 Center for Tuberculosis Control and Prevention, Xinjiang Uygur Autonomous Region Center for Disease Control and Prevention, Urumqi, Xinjiang, China; 3 Department of Respiratory Medicine, The First Affiliated Hospital of Xinjiang Medical University, Urumqi, Xinjiang, China; 4 Department of Traditional Uygur Medicine, Xinjiang Medical University, Urumqi, Xinjiang, China; 5 Research Institution of Health Affairs Development and Reform, Xinjiang Medical University, Urumqi, Xinjiang, China; FIOCRUZ, BRAZIL

## Abstract

**Objectives:**

Xinjiang is one of the high TB burden provinces of China. A spatial analysis was conducted using geographical information system (GIS) technology to improve the understanding of geographic variation of the pulmonary TB occurrence in Xinjiang, its predictors, and to search for targeted interventions.

**Methods:**

Numbers of reported pulmonary TB cases were collected at county/district level from TB surveillance system database. Population data were extracted from Xinjiang Statistical Yearbook (2006~2014). Spatial autocorrelation (or dependency) was assessed using global Moran’s I statistic. Anselin’s local Moran’s I and local Getis-Ord statistics were used to detect local spatial clusters. Ordinary least squares (OLS) regression, spatial lag model (SLM) and geographically-weighted regression (GWR) models were used to explore the socio-demographic predictors of pulmonary TB incidence from global and local perspectives. SPSS17.0, ArcGIS10.2.2, and GeoDA software were used for data analysis.

**Results:**

Incidence of sputum smear positive (SS+) TB and new SS+TB showed a declining trend from 2005 to 2013. Pulmonary TB incidence showed a declining trend from 2005 to 2010 and a rising trend since 2011 mainly caused by the rising trend of sputum smear negative (SS-) TB incidence (p<0.0001). Spatial autocorrelation analysis showed the presence of positive spatial autocorrelation for pulmonary TB incidence, SS+TB incidence and SS-TB incidence from 2005 to 2013 (*P* <0.0001). The Anselin’s Local Moran’s I identified the “hotspots” which were consistently located in the southwest regions composed of 20 to 28 districts, and the “coldspots” which were consistently located in the north central regions consisting of 21 to 27 districts. Analysis with the Getis-Ord Gi* statistic expanded the scope of “hotspots” and “coldspots” with different intensity; 30 county/districts clustered as “hotspots”, while 47 county/districts clustered as “coldspots”. OLS regression model included the “proportion of minorities” and the “per capita GDP” as explanatory variables that explained 64% the variation in pulmonary TB incidence (adjR^2^ = 0.64). The SLM model improved the fit of the OLS model with a decrease in AIC value from 883 to 864, suggesting “proportion of minorities” to be the only statistically significant predictor. GWR model also improved the fitness of regression (adj R^2^ = 0.68, AIC = 871), which revealed that “proportion of minorities” was a strong predictor in the south central regions while “per capita GDP” was a strong predictor for the southwest regions.

**Conclusion:**

The SS+TB incidence of Xinjiang had a decreasing trend during 2005–2013, but it still remained higher than the national average in China. Spatial analysis showed significant spatial autocorrelation in pulmonary TB incidence. Cluster analysis detected two clusters—the “hotspots”, which were consistently located in the southwest regions, and the “coldspots”, which were consistently located in the north central regions. The exploration of socio-demographic predictors identified the “proportion of minorities” and the “per capita GDP” as predictors and may help to guide TB control programs and targeting intervention.

## Introduction

Since the World Health Organization adopted the “declaration on the global TB emergency” in 1993, great achievements have been made over the past two decades. However, tuberculosis (TB) remains a major global health problem; in 2012, there were an estimated 8.6 million new TB cases and 1.3 million deaths due to tuberculosis [1]. This is a significantly large number of patients and deaths for a curable disease. The 22 high burden countries (HBCs) accounted for over 80% of the world`s TB cases, and China ranks second, accounting for 12% of global incidence [1]. From 2000 to 2010, the prevalence of active TB and sputum-smear positive (SS+) TB in China has declined from 466 /100000 to 459/100000 and from 169/100000 to 66/100000 respectively, according to the fifth national TB survey of 2010 [2]. However, the survey also reflected some problems existing in the current work of TB control in China, such as uneven distribution of TB cases. The prevalence of SS+ TB in the western area was 1.7 times that of the central region and 2.4 times that of the eastern region [2]. Xinjiang is one of the high TB burden provinces of China, in which there are more than 28,000 new TB cases and greater than 7,500 deaths each year [3]. The prevalence of active TB in Xinjiang has increased from 653/100000 in 2000 to 1526/100000 in 2010 according to the fifth national TB survey. Although the prevalence of sputum-smear positive (SS+) TB has decreased from 231/100000 in 2000 to 196/100000 in 2010, the current rate remains significantly higher than the national average. The prevalence of TB in Xinjiang also has large regional differences. Southern regions of Xinjiang have higher-TB burden regions, due in part to a less-developed economy and poverty [4].

Understanding such spatial variations in TB prevalence and its determinants within a social, spatial, and temporal context is crucial for improved targeting of interventions and resources. Geospatial analytical methods, such as geographic information systems (GIS), are essential tools for helping to achieve such understanding. There are increasing numbers of studies that use geospatial analytical methods in understanding TB or other public health problems [5–7], however, there are no related studies on geospatial distribution of TB in Xinjiang so far.

One reason for the limited use of GIS includes the scarcity of reliable, spatially-coded data. A web-based surveillance system has been applied for infectious disease surveillance in China since 2004, which has increasingly improved the efficacy and the speed of disease surveillance. Thus, the TB surveillance data in Xinjiang has been more reliable and accurate since 2005. Therefore, the main objectives of this study were to: (1) use GIS to analyze the TB surveillance data of Xinjiang from 2005 to 2013, (2) understand the geospatial characteristics of TB notification rates and (3) identify the social and demographic predictors of TB incidence.

## Methods

### 2.1 Data sources and variable definitions

Xingjiang Uyghur Autonomous region is the largest political subdivision of China, with an area of 1.66 million km^2^ and 22.33 million population in 2013. Xinjiang is divided into 14 prefectures (2 prefecture-level cities, 7 prefectures, and 5 autonomous prefectures). Then, these prefectures are further divided into 94 county/districts. Northern Xinjiang includes 7 prefectures, such as Urumqi, Karamay, Changji, Ili, Tarbagatay, Altay, and Bortala. Eastern Xinjiang includes 2 prefectures (Turpan and Kumul) and Southern Xinjiang includes 5 prefectures (Bayangol, Aksu, Kizilsu, Kashgar, and Hotan). The location of 94 county/districts is displayed in S1 Fig.

Numbers of reported TB cases for Xinjiang were collected at the county/district level from the internet-based National Infectious Diseases Reporting System (NIDRS), Chinese Center for Disease Control and Prevention. It is mandatory for all health care providers (hospitals, clinics, institutions of disease prevention and control and other designated health care establishments) to report all active pulmonary TB cases in a timely manner and directly via the NIDRS portal. The majority of reported TB cases are pulmonary, because reporting of pleural TB and extra-pulmonary TB is not mandatory. Therefore, analysis of this study was based on the pulmonary TB cases only.

The following four incidence rates were calculated by taking the population of each county/district in the same year as denominator respectively: the incidence of pulmonary TB, sputum smear positive TB (SS+TB, including sputum smear positive and/or culture positive), new SS+TB (SS+TB cases without TB treatment history) and sputum smear negative TB (SS-TB). Population data such as “population at year-end”, “male population”, “minority nationalities population”, “rural population”, “land area (sq.km)”, “death rate”, “per capita GDP (yuan)” were collected from the Xinjiang Statistical Yearbook, covering the years 2006 to 2014. In general, the yearbook is a record of information from the prior year Population density (population at year-end / land area), proportion of male (male population / population at year-end), proportion of minorities (minority nationalities population/ population at year-end), proportion of rural population (rural population / population at year-end), death rate and per capita GDP were analyzed as predictors of pulmonary TB incidence.

Ethics: The data from Statistical Yearbook are publicly available. The data from TB surveillance system were aggregated secondary data without any personal information and thus, informed consent was not needed. The study was approved by the Ethics Committee of The First Affiliated Hospital of Xinjiang Medical University.

### 2.2 Spatial analysis

#### 2.2.1 Spatial Autocorrelation Analysis

Spatial autocorrelation statistics have been commonly used to assess the degree of clustering, randomness or a fragmentation of a spatial pattern. Spatial autocorrelation includes global spatial autocorrelation which estimates the overall degree of spatial autocorrelation for a dataset, and the local spatial autocorrelation which identifies the location and types of clusters. The two most common spatial autocorrelation measures for continuous data are Moran`s I and Geary`s C statistics. Moran`s I is generally preferred over Geary`s C, because the values of the former are more intuitive (ie, positive values for positive autocorrelation and vice versa) [8]. Moran`s I was also found to be generally more robust [9]. Therefore, the global Moran`s I and Anselin`s Local Moran’s I statistics were respectively used to assess the global and local spatial autocorrelation of TB incidence in this study. Moran’s I statistics were calculated after log transformation of TB incidence to meet the criteria of normal distribution of the variable.

Global Moran’s I is computed as follows:

$$I=\frac{n\sum_{i=1}^{n}\sum_{j=1}^{n}w_{{}_{i,j}}z_{i}z_{j}}{S_{0}\sum_{i=1}^{n}z_{i}^{2}}$$

where Z_i_, Z_j_ are the deviations of an attribute for feature i and j from its mean $\left(x_{i}-\bar{X}\right)$ and $\left(x_{j}-\bar{X}\right)$, w_ij_ is the spatial weight between feature i and j, n is equal to the total number of features, and S_0_ is the aggregate of the all spatial weights $S_{0}=\sum_{i=1}^{n}\sum_{j=1}^{n}w_{{}_{i,j}}$.

Positive values indicate presence of positive spatial autocorrelation, which means the TB incidence should be similar among the neighboring districts comparing to the non-neighboring districts; zero means total spatial randomness; and negative values indicate dissimilar values clustered next to one another [10]. The absolute value of global Moran’s I indicates the strength of spatial autocorrelation. The statistical significance of Moran’s I is tested by Z score and *P* value. *P* <0.05 leads to rejection of the null hypothesis and indicates the presence of spatial autocorrelation.

Anslin`s Local Moran’s I is computed as follow:

$$I_{i}=\frac{x_{i}-\bar{X}}{S_{i}^{2}}\sum_{j=1,j\neq i}^{n}w_{i,j}\left(x_{j}-\bar{X}\right)$$

where x_i_ and x_j_ are the attributes for feature i and j, $\bar{X}$ is the mean of corresponding attribute, Wij is the spatial weight between feature i and j, n is equal to the total number of features, and $S_{i}^{2}=\frac{{\sum_{j=1,j\neq i}^{n}\left(x_{j}-\bar{X}\right)}^{2}}{n-1}-{\bar{X}}^{2}$.

A positive value for I indicates that a feature has neighboring features with similarly high or low attribute values; this feature is part of a cluster. A negative value for I indicates that a feature has neighboring features with dissimilar values. In either instance, the p-value for the feature must be small enough for the positive or negative spatial autocorrelation to be considered statistically significant. The types of spatial autocorrelation include “hotspots” (high values next to high, HH), “coldspots” (low values next to low, LL) and high amongst low, HL or vice versa, LH [11].

Presence of local clustering was further assessed using the Getis-Ord local statistic to provide additional information about the intensity and stability of core hotspot/coldspot clusters [12, 13]. The statistical significance of a Z-score assigned to each district identified the presence and intensity of local clusters of hotspots and coldspots of TB incidence, relative to the hypothesis of spatial randomness. The Getis-Ord Gi* index is calculated as:

$$G_{i}^{*}=\frac{\sum_{j=1}^{n}w_{i,j}x_{j}-\bar{X}\sum_{j=1}^{n}w_{i,j}}{S\sqrt{\frac{\left[n\sum_{j=1}^{n}w_{i,j}{}^{2}-{\left(\sum_{j=1}^{n}w_{i,j}\right)}^{2}\right]}{n-1}}}$$

where x_j_ is TB incidence for district j, w_ij_ is the spatial weight between districts i and j, n is the total number of districts (31), and

$$\bar{X}=\frac{\sum_{j=1}^{n}x_{j}}{n},\;S=\sqrt{\frac{\sum_{j=1}^{n}x_{j}^{2}}{n}-{\left(\bar{X}\right)}^{2}}.$$

#### 2.2.2 Spatial Regression Analysis

The study assumed that the predictors of pulmonary TB incidence have some lag effects. For example per capita GDP in 2005 was not only influencing the incidence of pulmonary TB in that year, but also affecting the incidence in the following years. Therefore, the study annualized the average values that were used when exploring the predictors of pulmonary TB incidence to increase the stability of data and minimize the potential bias [14]. The average incidences of pulmonary TB at each county/district over the 9-year-period were also calculated. This was helpful to alleviate the variation of incidence in small populations and districts.

Spatial data exhibits two properties that make it difficult to meet the assumptions and requirements of traditional (nonspatial) statistical methods, like ordinary least squares (OLS) regression [15]. One is spatial autocorrelation of variables, which makes it impossible to meet the criteria of independence of the data values. Another is non-stationary relations (spatial variation) of explanatory variables, which indicate varied behaviors in different parts of the study area. Global spatial regression models such as spatial lag model (SLM) and spatial error model (SEM) are used to effectively deal with the first characteristic (spatial autocorrelation) by isolating the spatial components of each input variable and put it back into the regression model as a new variable to account for spatial effects. However, those global models could not explore the spatial variation in the relations between TB incidence and the predictors. Geographically-weighted regression (GWR) model, however, could be used to explore spatial variation (nonstationarity) in the relations and provide more detailed information. GWR is a local regression model which creates an equation for every feature (each county/district, in this case) and calibrates with it using nearby features. The closer features have a larger impact on calibration than features that are further away. Because each feature has its own equation, coefficients are allowed to vary over space [16]. GWR model has been increasingly used in spatial epidemiology [17, 18].

Although the OLS model is not proper for spatial data, it is always the proper starting point for all spatial regression analyses. In the first step, OLS regression was conducted and results were evaluated according to the OLS requirements [19]: coefficients for model explanatory variables should be statistically significant and have the expected sign (+/-); explanatory variables must be free from multicollinearity; the model should not be biased (heteroscedasticity or non-stationarity); residuals must be normally distributed with a mean of zero; the model cannot be missing key explanatory variables; and residuals must be free from spatial autocorrelation [15]. In the next step, SLM was chosen as the global spatial regression model based on Lagrange Multiplier (LM) test statistics [20]; both LM-Lag and LM-Error are statistically significant. Of the robust forms, only the Robust LM-Lag statistic is statistically significant (*p* < 0.01), while the Robust LM-Error statistic is not (p = 0.90). SLM directly incorporates spatial autocorrelation into the model by including a spatial lag term (ρ). Formally, this model is y = ρWy+Xβ+ε, where y is a vector of observations on the dependent variable, Wy is a spatially lagged dependent variable for weights matrix W, X is a matrix of observations on the independent variables, ε is a vector of error terms, ρ and β are regression coefficients. In the third step, GWR was used as local spatial regression model to explore the spatial variation in the relations between pulmonary TB incidence and predictors. In this method, the critical part lies in the optimal bandwidth allocation. This paper chose the Akaike Information Criterion (AIC), which was fixed by the maximum likelihood principle to determine the optimal bandwidth. Moran's Index was used to test spatial autocorrelation of residuals. Log-Likelihood and AIC value were used to compare the fitness of OLS and SLM [20], while adjusted R^2^ and AIC value were used to compare the fitness of OLS and GWR [21].

The software SPSS 17.0, ArcGIS 10.2.2 (ESRI Inc., Redlands, CA, USA) and GeoDa (https://geodacenter.asu.edu/software) were used for data analysis. County boundary electronic map of Xinjiang Autonomous Region at 1:100,000 scale was intercepted from a county boundary map of China from the National Geographic Information System database (http://nfgis.nsdi.gov.cn).

## Results & Discussion

### 3.1 Trend of pulmonary TB incidence from 2005 to 2013

**Fig 1:**
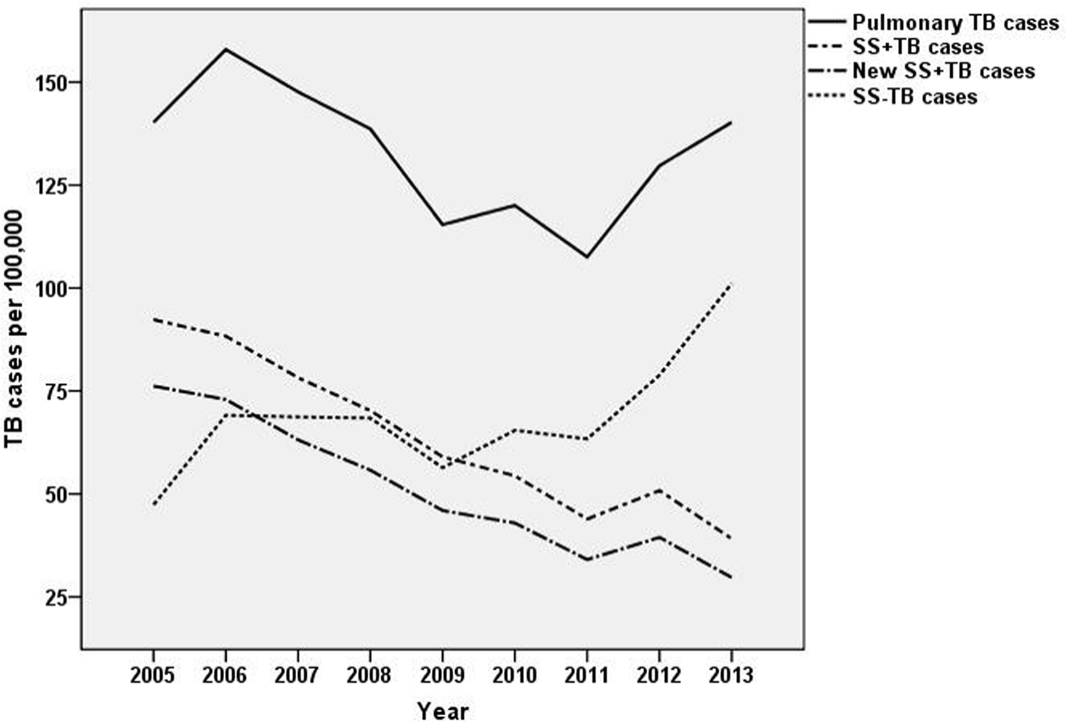
The trend of pulmonary TB incidence from 2005 to 2013.

The annualized average incidences of pulmonary TB, SS+TB, and new SS+TB over the 9 year-period were 132.59, 63.17, and 50.36 per 100,000 respectively. From 2005 to 2013, the incidences of SS+TB and the new SS+TB have decreased respectively from 92/100,000 to 39/100,000 and from 76/100,000 to 30/100,000. Both of the SS+TB and new SS+TB incidence rate demonstrated a statistically significant trend in decline from 2005 to 2013 (χ^2^_linear_ = 8896 and 8471 respectively, *P*<0.0001). The SS-TB incidence was relatively stable from 2005 to 2010 and showed a statistically significant rising trend since 2011 (χ^2^_linear_ = 1894, p<0.0001). The statistically significant trend in decline of pulmonary TB incidence (χ^2^_linear_ = 1211, p<0.0001) from 2005 to 2011was mainly caused by the decline of SS+TB cases, while the rising trend of pulmonary TB incidence since 2011 (χ^2^_linear_ = 914, p<0.0001)was due to the increase of SS-TB cases (Fig 1).

The dramatic decline of SS+TB and new SS+TB incidence reflects the effectiveness of TB control and implementation of DOTS strategy over the past two decades. However, the TB incidence rates of Xinjiang were still significantly higher than the national average for each year [22]. For example, the incidence rates of total TB and SS+TB in China were 78.1 and 31.9 per 100,000 in 2011 [22], while these incidences in Xinjiang, in the same year, were 108.1 and 43.9 per 100,000 respectively. According to the results of fifth national TB survey in 2010, the prevalence of total TB and SS+TB in Xinjiang was three times the value of the national average prevalence [4]. Therefore, TB control in Xinjiang remained an arduous task.

The rising trend of pulmonary TB incidence since 2011 which is caused by the increase of SS-TB incidence is mainly due to the standardized diagnosis and improved notification of SS-TB in recent years, especially since the fifth national TB survey in 2010. Previously, the focus was on the control of SS+TB relative to SS-TB in the initial implementation of many TB control projects to control the main source of infection. However, SS-TB patients also play a great role in TB epidemic which accounts for 40~60% of all TB cases and half of them will be converted to SS+TB without treatment [23]. Therefore, recently the government is making better efforts to improve the job of diagnosing and reporting of SS-TB cases.

### 3.2 Distribution of pulmonary TB incidence at district/county level

**Fig 2:**
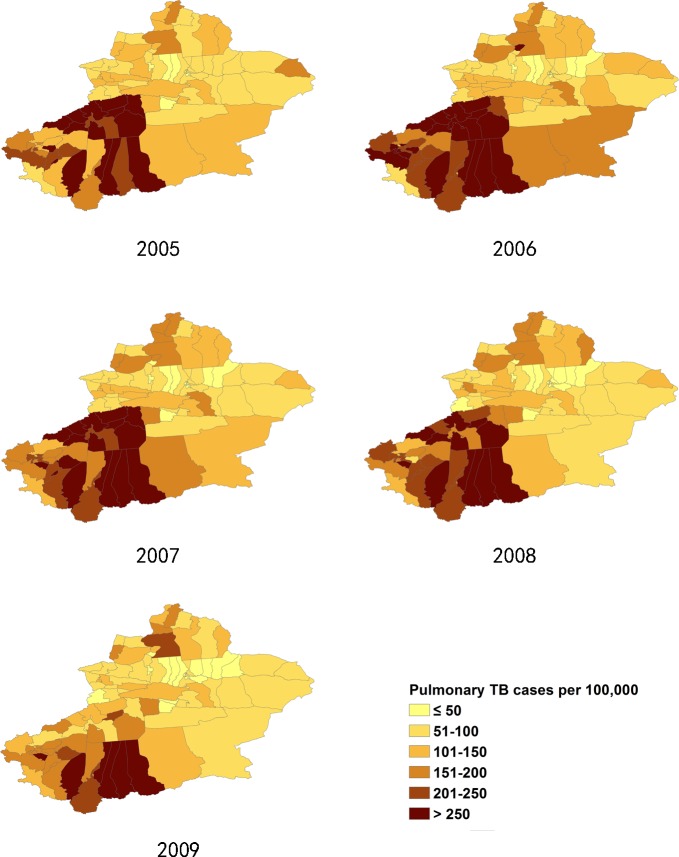
The incidence of pulmonary TB cases in Xinjiang, from 2005–2009.

**Fig 3:**
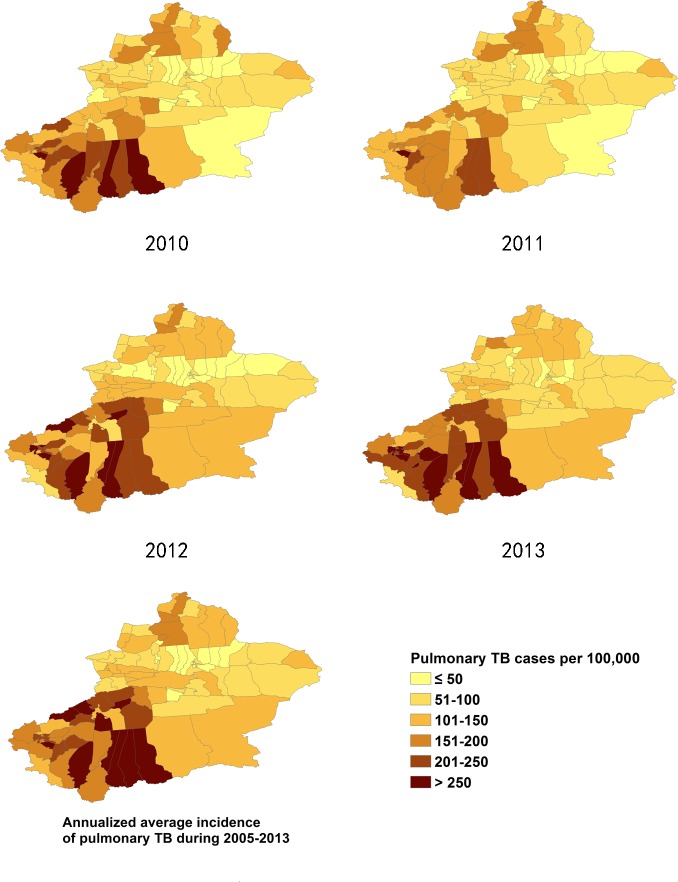
The incidence of pulmonary TB cases in Xinjiang, from 2010–2013.

**Fig 4:**
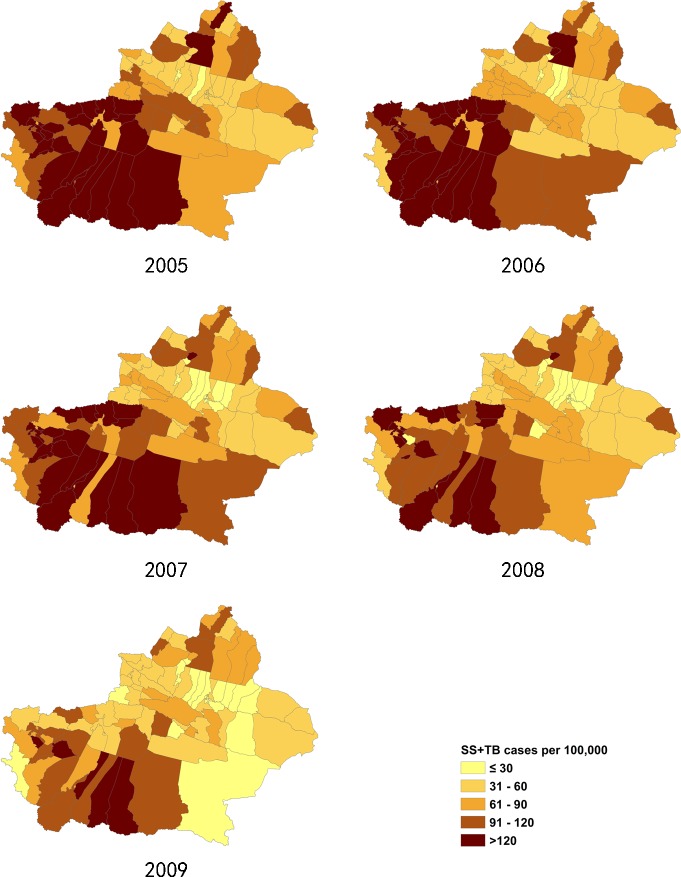
The incidence of SS+TB cases in Xinjiang, from 2005–2009.

**Fig 5:**
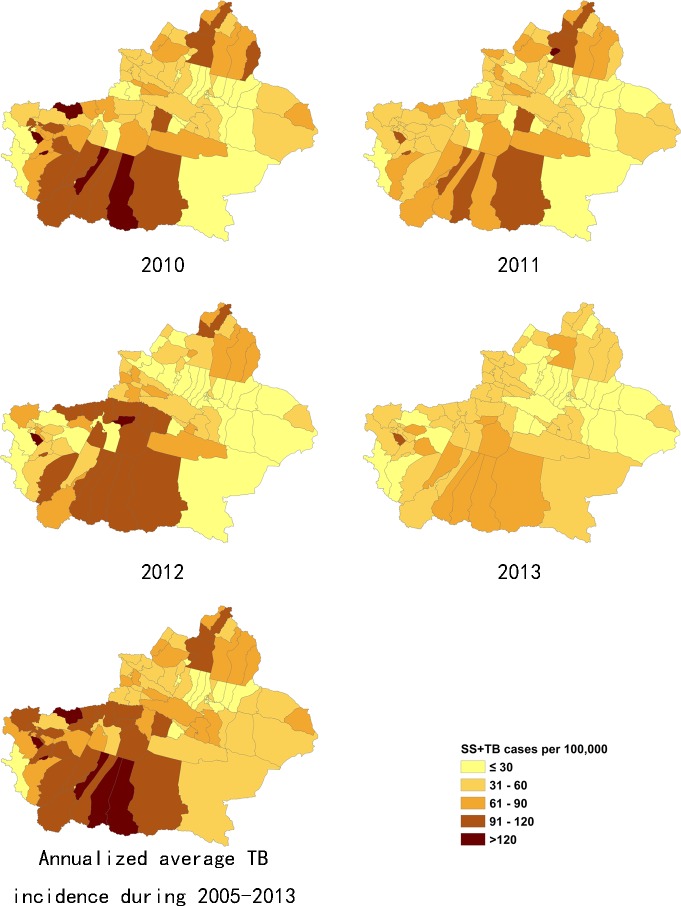
The incidence of SS+TB cases in Xinjiang, from 2010–2013.

The incidence of pulmonary TB in Xinjiang had large regional differences. The ranges of annualized average incidence of pulmonary TB and SS+TB were 17 to 338/100,000 and 10 to 168/100,000 respectively. The pulmonary TB incidence of the highest TB burden counties was 15 to 20 times of the incidence of the lowest TB burden counties. In general, the southern Xinjiang (southern part of Tianshan mountains), especially the southwest part of Xinjiang, had a high incidence of pulmonary TB and SS+TB. Most of the counties of Aksu, Hotan, Kashgar and Kizilsu prefectures had more than 150/100,000 pulmonary TB incidence and more than 90/100,000 SS+TB incidence. On the other hand, some counties at the northern end of Xinjiang, such as Jemnay County, Burqin County, Hoboksar Mongol Autonomous County and Urhe District had higher TB incidence rates. (Figs 2–5).

### 3.3 Global spatial autocorrelation analyses

**Fig 6:**
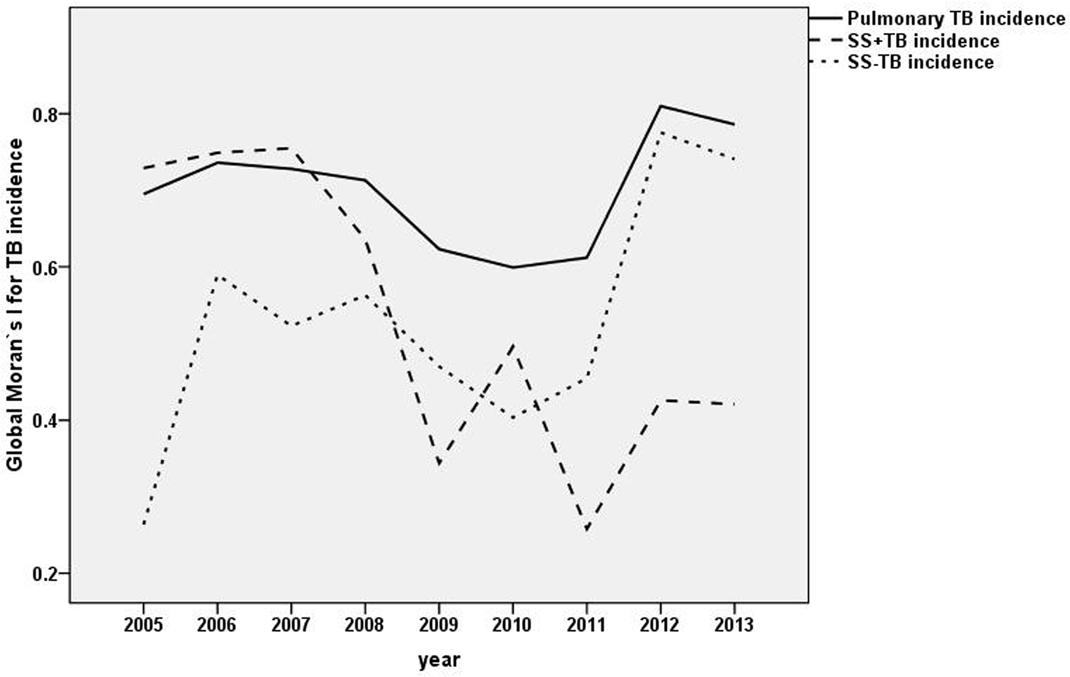
The trend of Global Moran`s I value for pulmonary TB incidence from 2005 to 2013.

The global spatial autocorrelation analysis showed the presence of positive spatial autocorrelation (global Moran’s I > 0) in pulmonary TB, SS+TB and SS-TB incidence from 2005 to 2013 (*P* <0.0001, see Table 1). There was a significant temporal variation in the spatial autocorrelation of SS+TB incidence, the absolute value of global Moran’s I has decreased from 2005~2013 (t = -0.811, *P* = 0.008). However, there was no statistically significant trend on the global Moran’s I of pulmonary TB and SS-TB incidence (t = 0.145 and 0.618 respectively, *P* >0.05) (Fig 6).

**Table 1:** Results of the global spatial autocorrelation analysis of pulmonary TB incidence from 2005–2013.

Year	Pulmonary TB incidence			SS+ TB incidence			SS- TB incidence		
	Moran`s I	Z	*P*	Moran`s I	Z	*P*	Moran`s I	Z	*P*
2005	0.695	18.497	<0.0001	0.729	19.389	<0.0001	0.264	7.132	<0.0001
2006	0.736	19.328	<0.0001	0.749	19.712	<0.0001	0.590	15.560	<0.0001
2007	0.728	19.130	<0.0001	0.755	19.873	<0.0001	0.523	13.821	<0.0001
2008	0.713	18.746	<0.0001	0.636	16.757	<0.0001	0.563	14.847	<0.0001
2009	0.623	16.429	<0.0001	0.344	9.464	<0.0001	0.470	12.525	<0.0001
2010	0.599	15.818	<0.0001	0.496	13.088	<0.0001	0.404	10.804	<0.0001
2011	0.612	16.108	<0.0001	0.258	7.026	<0.0001	0.455	12.148	<0.0001
2012	0.810	21.228	<0.0001	0.426	11.327	<0.0001	0.775	20.336	<0.0001
2013	0.786	20.634	<0.0001	0.421	11.013	<0.0001	0.741	19.449	<0.0001
Annualized average incidence	0.78	20.733	<0.0001	0.717	18.843	<0.0001	0.696	18.277	<0.0001

This result is consistent with other studies, in which spatial analysis of global TB distribution [24] or national distribution [25], even the distribution in a city [26] also showed significant spatial autocorrelation. Therefore understanding the spatial characteristics of TB distribution provides useful information for the development of more targeted TB control policies. One thing worth mentioning is that Moran’s I value of SS+TB had a significant declining trend from 2005 to 2013. The absolute value of global Moran’s I indicates the strength of spatial autocorrelation. And a decrease in strength of spatial autocorrelation for SS+TB suggests a decrease in regional differences of SS+TB. Reducing the regional difference of disease distribution and achieving health equity is one of the goals of public health effort. This may be partially due to the free treatment policy for SS+TB patients and greater attention given by the government to SS+TB control. However, such a trend did not appear in pulmonary TB and SS-TB incidence. Therefore more importance should be attached to the SS-TB control especially in the high TB burden counties.

### 3.4 Positive and negative spatial autocorrelation of pulmonary TB incidence

**Fig 7:**
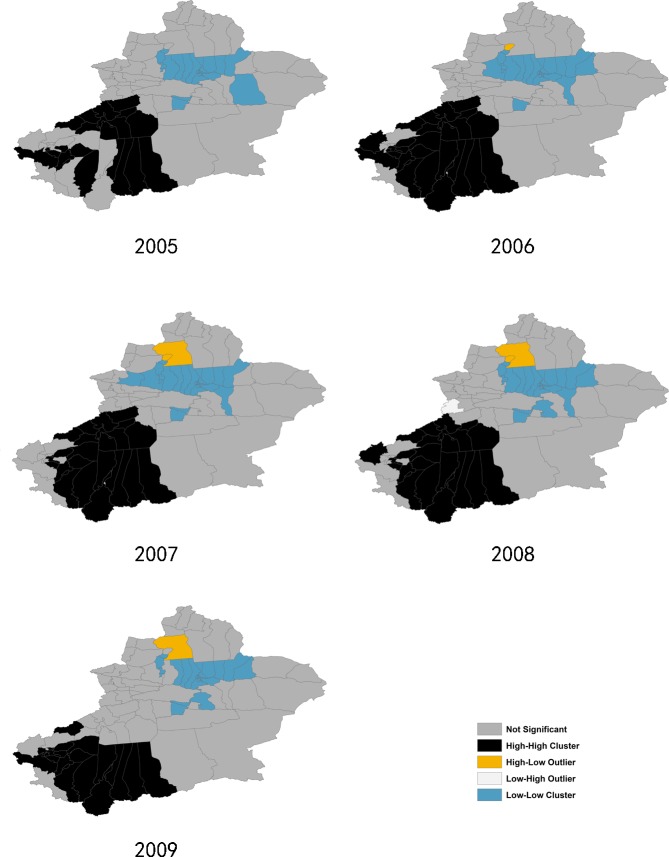
Clusters of the Anselin Local Moran’s I analysis, from 2005–2009.

**Fig 8:**
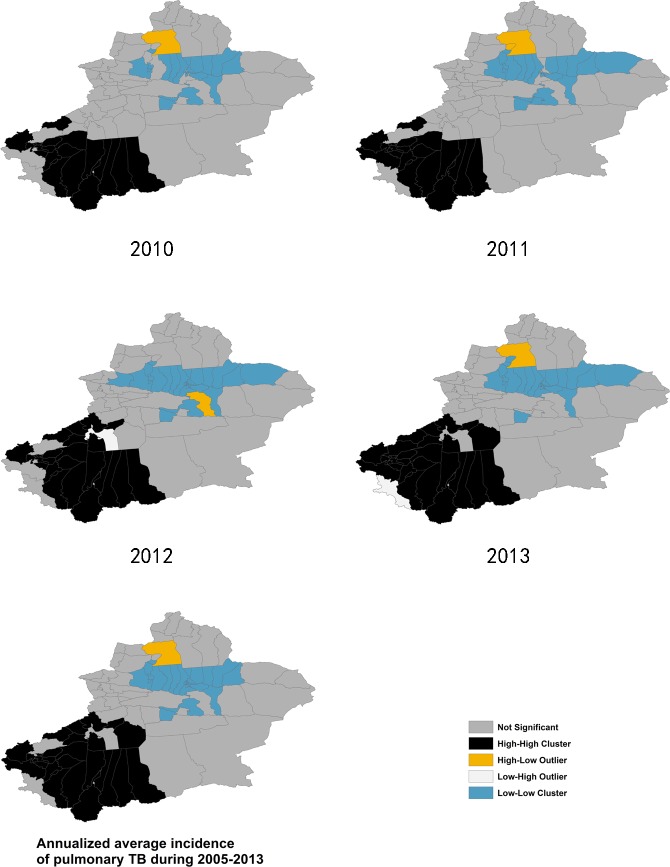
Clusters of the Anselin Local Moran’s I analysis, from 2010–2013.

Local spatial analysis revealed a statistically significant clustering of districts into ‘hotspots’ and ‘coldspots’ of pulmonary TB incidence, showing a significant change over time (Figs 7 and 8). The Anselin’s Local Moran’s I showed that the core clustering of high TB incidence districts next to high ones (HH) consistently located in the southwest regions composed of 20 to 28 districts from 2005 to 2013. HH clusters in 2005 to 2008 included most of the counties of Aksu, Hotan Prefecture, some counties of Kashgar and Kizisu Kirgiz Autonomous Prefecture. Since 2009, HH clusters moved toward southwest more obviously, including most of the counties of Kashgar, Hotan and Kizisu Kirgiz Autonomous Prefecture, no longer or partly including the counties of Aksu Prefecture.

Analysis also showed a core “coldspot” cluster of low-next-to-low (LL) districts consistently located in the north central regions consisting of 21 to 27 districts. LL clusters included all districts of Urumqi City, Chanji Hui Autonomous Prefecture, Karamay City (except Dushanzi district), some counties in northern part of Bayangol Mongol Autonomous Prefecture, Usu City, Turpan City, and Shawan County.

Statistically significant spatial outliers (HL, LH clustering) were evident only for a few years. Urhe District of Karamay City had exceptionally high TB incidence next to a low incidence neighborhood (HL cluster) from 2006 to 2008 and 2011, because a small population (about 2,000) of the district caused the big variation in TB incidence. Hoboksar Mongol Autonomous County had also been a HL cluster from 2007 to 2011 and 2013. Mongolkure County, Aksu City and Taxkorgan Tajik Autonomous County had a significantly low incidence surrounded by high incidence districts (LH cluster) only in 2008, 2012, and 2013, respectively.

**Fig 9:**
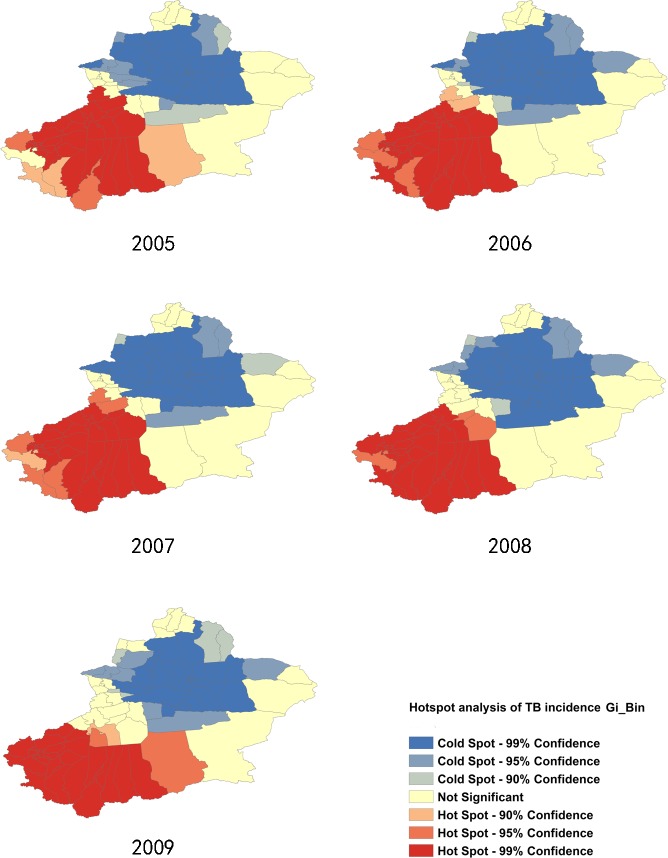
Hotspot Analysis with Getis-Ord Gi* statistic, from 2008–2009.

**Fig 10:**
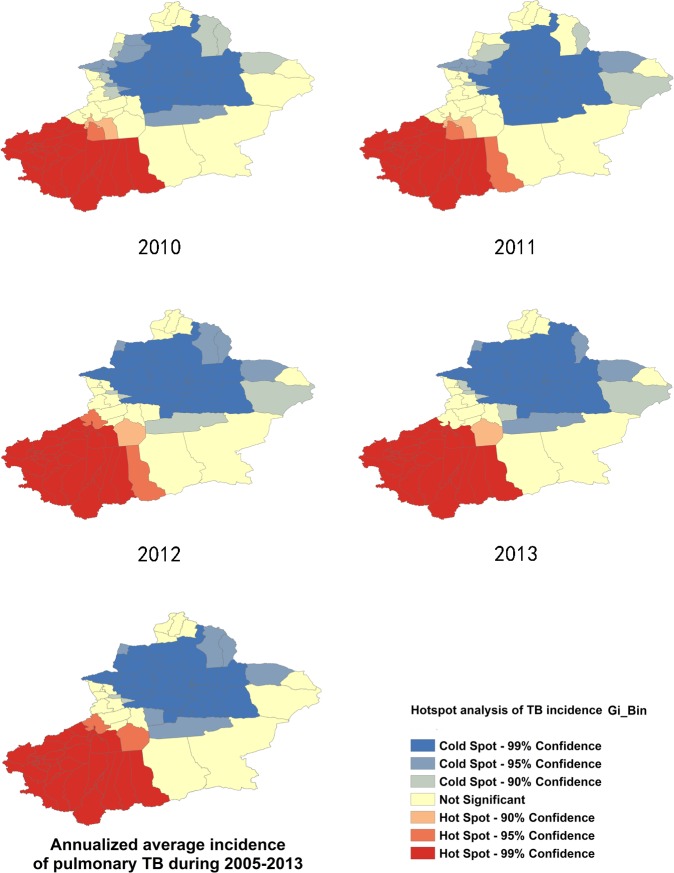
Hotspot Analysis with Getis-Ord Gi* statistic, from 2010–2013.

Analysis with the Getis-Ord Gi* statistic provided more information that indicates the intensity and the stability of core hotspot/coldspot clusters. Primary (GiZScore>2.58 SDs), secondary (GiZScore = 1.96–2.58 SDs), and tertiary (GiZScore = 1.64–1.96 SDs) intensity clusters from 2005 to 2013 are presented in Figs 9 and 10. Hotspot/coldspot clusters of annualized average incidence of pulmonary TB are described in Table 2.

**Table 2:** Summary of hotspot/coldspot clusters of annualized average incidence of pulmonary TB in Xinjiang.

Types of clusters	Intensity of clusters	Numbers of counties	County/districts
Hotspots	Primary	28	Aksu City, Uxturpan County, Awat County, Kalpin County, Artux City, Akto County, Akqi County, Ulugqat County, Kashgar City, Shufu County, Shule County, Yengisar County, Poskam County, Yarkent County, Kagilik County, Makit County, Yopurga County, Payzawat County, Maralbexi County, Taxkorgan Tajik Autonomous County, Hotan City, Hotan County, Karakax County, Guma County, Lop County, Qira County, Keriya County, Niya County
	Secondary	2	Onsu County, Xayar County
Coldspots	Primary	40	Tianshan District, Shayibak District, Xinshi District, Shui Mogou District, Tou Tunhe District, Da Bancheng District, Midong District, Urumqi County, Dushanzi District, Karamay District, Urhe District, Bai Jiantan District, Changji City, Fukang City, Hutubi County, Manas County, Qitai County, Jimsar County, Mori Kazak Autonomous County, Turpan City, Piqan County, Toksun County, Kuytun City, Kunes County, Nilka County, Bortala City, Jing County, Araxang County, Usu City, Dorbiljin County, Shawan County, Toli County, Yumin County, Hoboksar Mongol Autonomous County, Burultokay County, Korla City, Yanji Hui Autonomous County, Hejing County, Hoxud County, Bagrax County
	Secondary	6	Lopnur County, Bugur County, Qinggil County, Koktokay County, Qoqak City, Barkol Kazak Autonomous County.
	tertiary	1	Tokkuztara County,

Hotspot analysis was conducted separately for SS+TB and SS-TB incidences. Hotspot/coldspot clusters for SS+TB and SS-TB incidence were almost at the same location with pulmonary TB incidence. Hotspots located in the southwest region included Hotan, Kashgar, and Kizilsu Kirgiz Autonomous Prefecture, while coldspots were located in the north central regions (S2–S5 Figs).

The analysis using the Getis-Ord Gi* statistic expanded the scope of the “hotspots” and the “coldspots” with different intensities. Thirty county/districts clustered as “hotspots”, while 47 county/districts clustered as “coldspots”. These two big clusters seem to be separated by Tianshan Mountains in Xinjiang. Xinjiang is the largest political subdivision of China, accounting for more than one sixth of China's total territory and a quarter of its boundary length. It is divided into two basins by Tianshan Mountains, Dzungarian Basin in the north and Tarim Basin in the south. Southern Xinjiang is higher in temperature and lower in precipitation compared to the northern regions because of the Taklimakan Desert, which is China's largest desert located in the center of the Tarim Basin south of the Tianshan Mountains. Climate differences between northern and southern Xinjiang influence the agricultural products, which contributes to the poverty in southern Xinjiang. Poverty is one of the possible reasons apart from climate factors, lead to high TB incidence in southern Xinjiang. Additional factors leading to high TB incidence include: underdeveloped economy, poor traffic conditions, and uneven allocation of public health resources. Taking traffic conditions for example, railway transportation to Hotan was not possible until 2011 [27]. Another important reason may be the fact that the southwest regions of Xinjiang border some of the high-TB burden countries like Afghanistan, Pakistan, and India. Therefore, the high TB burden of those countries may also affect the southwest parts of Xinjiang. Some studies showed that different genotypes of Mycobacterium tuberculosis have different virulence and transmission advantages [28]. Thus, the different strains of Mycobacterium that cause tuberculosis in different regions are a possible explanation for regional differences of pulmonary TB incidence.

### 3.5 Socio-demographic predictors of pulmonary TB incidence

Six independent variables such as “population density”, “proportion of male”, “proportion of minorities”, ‘proportion of rural population”, “death rate” and “per capita GDP” were selected according to our review of the relevant epidemiological literature and the availability of relevant data at county/district level. Two variables were found to be statistically significant predictors for pulmonary TB incidence in OLS regression model. The “proportion of minorities” had a positive effect on pulmonary TB incidence, while “per capita GDP” had a negative effect. This model met most of the requirements of the OLS method: the robust probabilities for the explanatory variable coefficients were statistically significant (*P* < 0.05); Variance inflation factor (VIF) values were low (VIF< 2.0) indicating no problems with multicollinearity; The Joint Wald Statistic indicated overall model significance (*P* < 0.01); The non-significant (*P* > 0.10) Jarque-Bera diagnostic indicated model residuals were normally distributed. However, statistical significant autocorrelation in residuals (Moran`s I = 0.12, Z = 3.23, *P* = 0.0012) indicated the spatial autocorrelations of variables and the non-stationary nature of OLS regression model.

Spatial Lag Model (SLM) was used as global spatial regression model to deal with the spatial autocorrelation of variables. The SLM results showed positive spatial autocorrelation of pulmonary TB incidence (ρ = 0.3881, *P* <0.01); The “proportion of minorities” had a positive effect on TB incidence in the SLM model (β = 1.7004, *P* <0.01), and the absolute value of the regression coefficient was decreased compared to the OLS model. The regression coefficient of the “per capita GDP” no longer showed statistical significance (*P* >0.05) in the SLM model. The decreasing values of Log-Likelihood and AIC also suggested the improvement of the model fit in SLM compared to OLS (see Table 3). Moran’s I test statistic for residuals of the SLM model was 0.0009, or essentially zero. This indicates that including the spatially lagged dependent variable term in the model has eliminated all spatial autocorrelation, as it should.

**Table 3:** Summary of OLS and SLM regression model.

Model	Parameter	Coefficient	Std.Error	t / z	Probability	Log-Likelihood	AIC
OLS	Constant	11.2189	24.8464	0.45	0.65	-434.55	883.89
	Proportion of minorities	2.1573	0.2593	8.32	<0.01		
	per capita GDP	-0.0005	0.0003	-2.06	0.04		
SLM	Wy	0.3881	0.0946	4.10	<0.01	-427.61	864.48
	Constant	-22.5312	23.3347	-0.96	0.33		
	Proportion of minorities	1.7004	0.2583	6.58	<0.01		
	per capita GDP	-0.0002	0.0003	-0.49	0.62		

A GWR model was used as local spatial regression model to deal with the non-stationary nature of the OLS model. The GWR model improved the fit of OLS model with an increase of adjusted R^2^ from 0.64 to 0.68 and a decrease of AIC value from 883.89 to 871.47. AIC is an effective way to compare models, and a drop of even three points indicates an important improvement in model fit. Although the model fit of GWR is weaker than SLM according to AIC value, it was concerned the spatial variation of coefficient estimation. But the GWR model also has a drawback that it depends on the calculation of distance weights which is very arbitrary for polygon-support data. The summary of the GWR model results was listed in Table 4.

**Table 4:** Summary of GWR model coefficients.

Parameter	Minimum	25% quartile	50% quartile	75% quartile	Maximum
Intercept	-32.69	-14.66	1.76	11.32	139.38
Proportion of minorities	1.07	1.76	2.01	2.39	2.68
Per capita GDP	-0.0027	-0.0001	0.0000	0.0001	0.0002
Condition number	6.39	7.25	7.81	13.49	29.99
Local R^2^	0.2506	0.5271	0.5972	0.6366	0.6940

**Fig 11:**
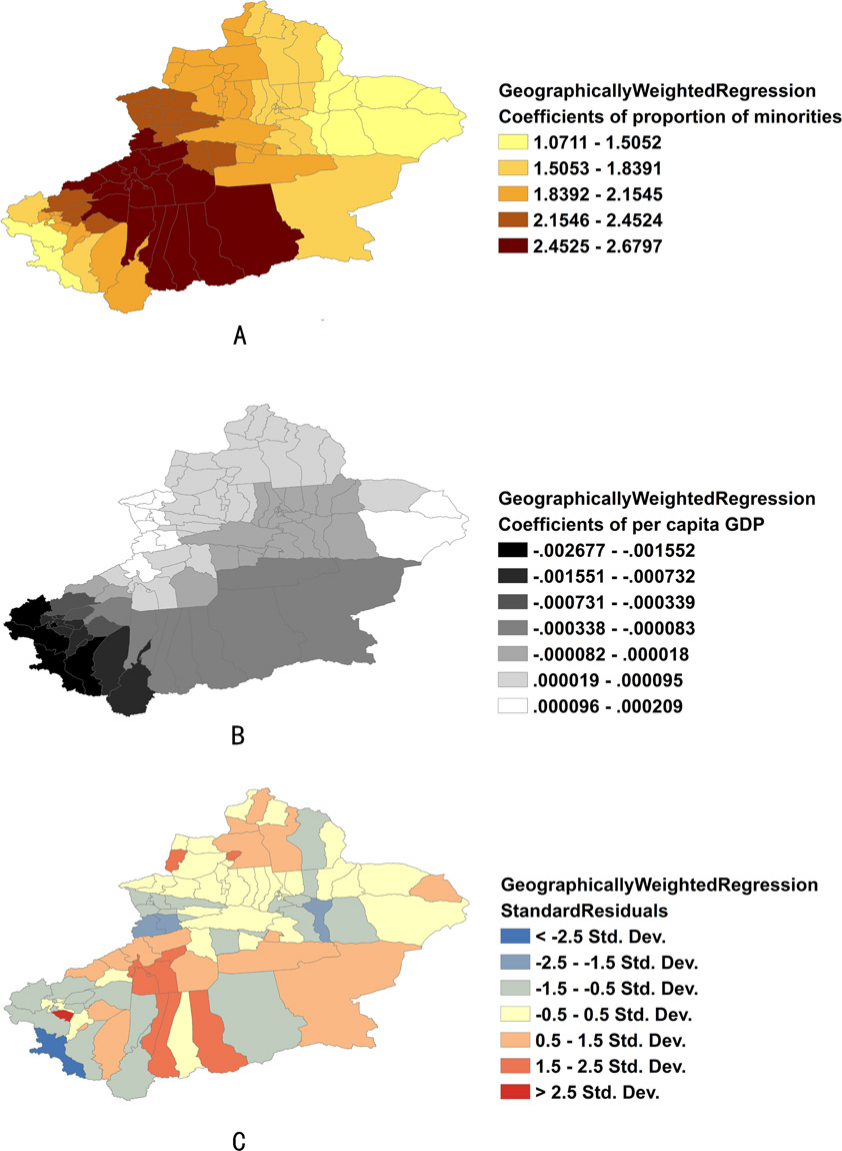
Coefficients of predictors and standard residuals of GWR. (A) Coefficients of proportion of minorities. (B) Coefficients of per capita GDP. (C) Standard residual of GWR.

The mapped coefficients for each county/districts (Fig 11) indicated where the explanatory vaables were effective predictors of pulmonary TB incidence and where they were not. In Fig 11, the counties rendered using the darkest colors indicate where the coefficient is the largest. The larger the coefficient is, the stronger the relationship is. Proportion of minorities, for example, had a positive relationship to pulmonary TB incidence: as the proportion of minorities increased, pulmonary TB incidence also increased. The “proportion of minorities” is a strong predictor in the south central counties (Fig 11A). Per capita GDP had a negative relationship to pulmonary TB incidence: as the per capita GDP increased, pulmonary TB incidence decreased. Per capita GDP is a weak predictor in the eastern regions where the coefficient is near zero and even slightly positive; however it is a strong predictor for the southwest regions (Fig 11B). Standard residual of GWR was also mapped in Fig 11C. Red colors indicated underestimation of pulmonary TB incidence according to the regression equation while the blue colors indicated overestimation. Under/overestimated counties were randomly located and the Moran’s I for residuals of GWR model is not significant (*P* >0.05).

OLS, SLM and GWR models were used to explore the predictors of pulmonary TB incidence. Results showed that the SLM and GWR models had a better fitness than the OLS model. Proportion of minorities had a positive relationship to TB incidence and it was a strong predictor especially in the south central counties. The minorities were lower in social status and economic level. Therefore, the proportion of those populations had a positive correlation with pulmonary TB incidence. Those results are consistent with other studies [29–31]. Per capita GDP had a negative relationship to pulmonary TB incidence and it was a strong predictor for the southwest regions. As we know TB is a poverty related disease, so it is not necessary to explicitly explain the negative correlation of per capita GDP with TB. However, both of the “proportion of minorities” and “per capita GDP” included in the regression model as significant factors indicated that minorities were high risk population without the confounding of their economic status. Genetic factors could be an explanation because results of some studies showed the genetic susceptibility to TB in minorities [32–34].

Based on the results of this study, we suggest that the government’s future efforts in TB control should give more priorities to southern Xinjiang. Providing free treatment for all TB patients (not only for SS+TB patients) in southwest regions could be considered when making public health policies, because the economic status was a strong predictor of the pulmonary TB incidence in the southwest regions. Furthermore, it is necessary to conduct research on the genetic susceptibility of minorities, in particular the Uygur population, which is the major population in southern Xinjiang.

## Limitations

This study contributed significantly to spatiotemporal analysis of TB incidence of Xinjiang, yet it also has limitations. First, the data was extracted from official surveillance which cannot exclude the possibility of underreport of cases in some regions. Cases may be missed by routine notification systems, because persons afflicted with TB often do not seek care or if they seek care, remain undiagnosed or are diagnosed by private providers that do not report TB cases to local or national authorities. Second, this is an ecological study which is exploring the association at a group level, so ecological fallacy is the critical limitation of this study. The best solution to the ecologic fallacy is multi-level modelling which includes both individual and ecological-level variables [35]. However this study uses only the ecological variables in the model. When studying the social and ecological factors, ecological studies were preferred compared to non-ecological studies and ecological fallacy can be weakened by combining secular variations [36]. In this regard, the present study was conducted to explore the socio-demographic factors for the spatial heterogeneity of pulmonary TB incidence by annualized average values of variables. Thirdly, the ecological factors related to pulmonary TB incidence have not been well studied because of unavailability of many factors at county/district level. We intended to widely explore the predictors of TB incidence, including demographic variables (such as population density, proportion of rural population, proportion of minorities…), economic factors (such as per capita GDP, per capita disposable income…), climate factors (annual average temperature, annual total precipitation…) and health related factors (death rate, numbers of beds/doctors per 10,000…). However, most of the variables were available only at prefecture-level (n = 14), not at county/district level (n = 94). It is not proper to conduct regression analysis for so many factors in a small sample size (14 prefecture divisions). Thus we analyzed only six variables which were available at county/district level, and found two important factors. The adjusted R^2^ of regression model was 0.64 for OLS and 0.68 for GWR model, which means those two variables can explain more than sixty percent variations of pulmonary TB incidence. Further research is needed to study the socio-economic and environmental factors, with a focus on those factors that can be affected by intervention, and this will be more conductive to guide policy formulation.

## Conclusions

SS+TB incidence of Xinjiang had a decreasing trend from 2005 to 2013, but remains higher than the national average in China. Spatial analysis showed that all of the pulmonary TB incidence, SS+TB incidence and SS-TB incidence had a significant spatial autocorrelation each year. Cluster analysis detected two clusters, the “hotspots” were consistently located in the southwest regions and the “coldspots” were consistently located in the north central regions. The exploration of socio-demographic predictors identified two predictors, “proportion of minorities” that had a positive correlation with pulmonary TB incidence and “per capita GDP” that had a negative correlation with pulmonary TB incidence. Proportion of minorities was a strong predictor in the south central counties, while per capita GDP was a strong predictor for the southwest regions. Therefore, we suggest that the government’s future efforts in TB control should give more priorities to southern Xinjiang. Providing free treatment for all TB patients (not only for SS+TB patients) in southwest regions could be considered when making public health policies, because the economic status was a strong predictor of the TB incidence in the southwest regions. Furthermore, it is necessary to conduct research on the genetic susceptibility of minorities, in particular the Uygur population, which is the major population in southern Xinjiang.

## Supporting Information

S1 Fig County boundary electronic map of Xinjiang Uyghur Autonomous Region. (TIF)

S2 Fig Hotspot Analysis with Getis-Ord Gi* statistic for SS+TB, from 2008–2009. (TIF)

S3 Fig Hotspot Analysis with Getis-Ord Gi* statistic for SS+TB, from 2010–2013. (TIF)

S4 Fig Hotspot Analysis with Getis-Ord Gi* statistic for SS-TB, from 2008–2009. (TIF)

S5 Fig Hotspot Analysis with Getis-Ord Gi* statistic for SS-TB, from 2010–2013. (TIF)
