# Supplementary figures and images for: Socio-Demographic Predictors and Distribution of Pulmonary Tuberculosis (TB) in Xinjiang, China: A Spatial Analysis
Source: PLoS One. 2015 Dec 7;10(12):e0144010. doi: 10.1371/journal.pone.0144010 (PMC4671667; doi:10.1371/journal.pone.0144010)

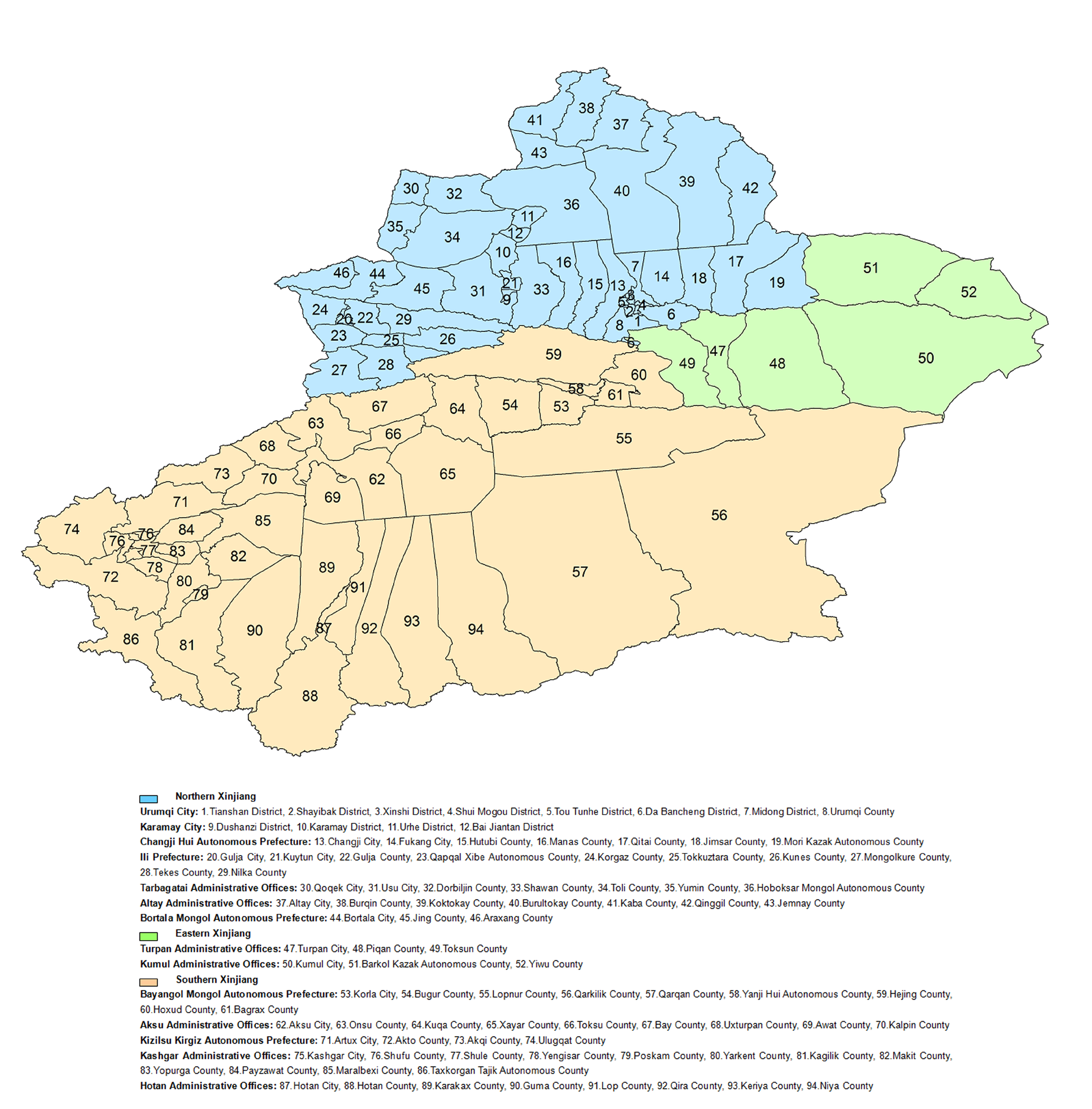

Supplement: S1 Fig — (TIF) [file pone.0144010.s001.TIF]

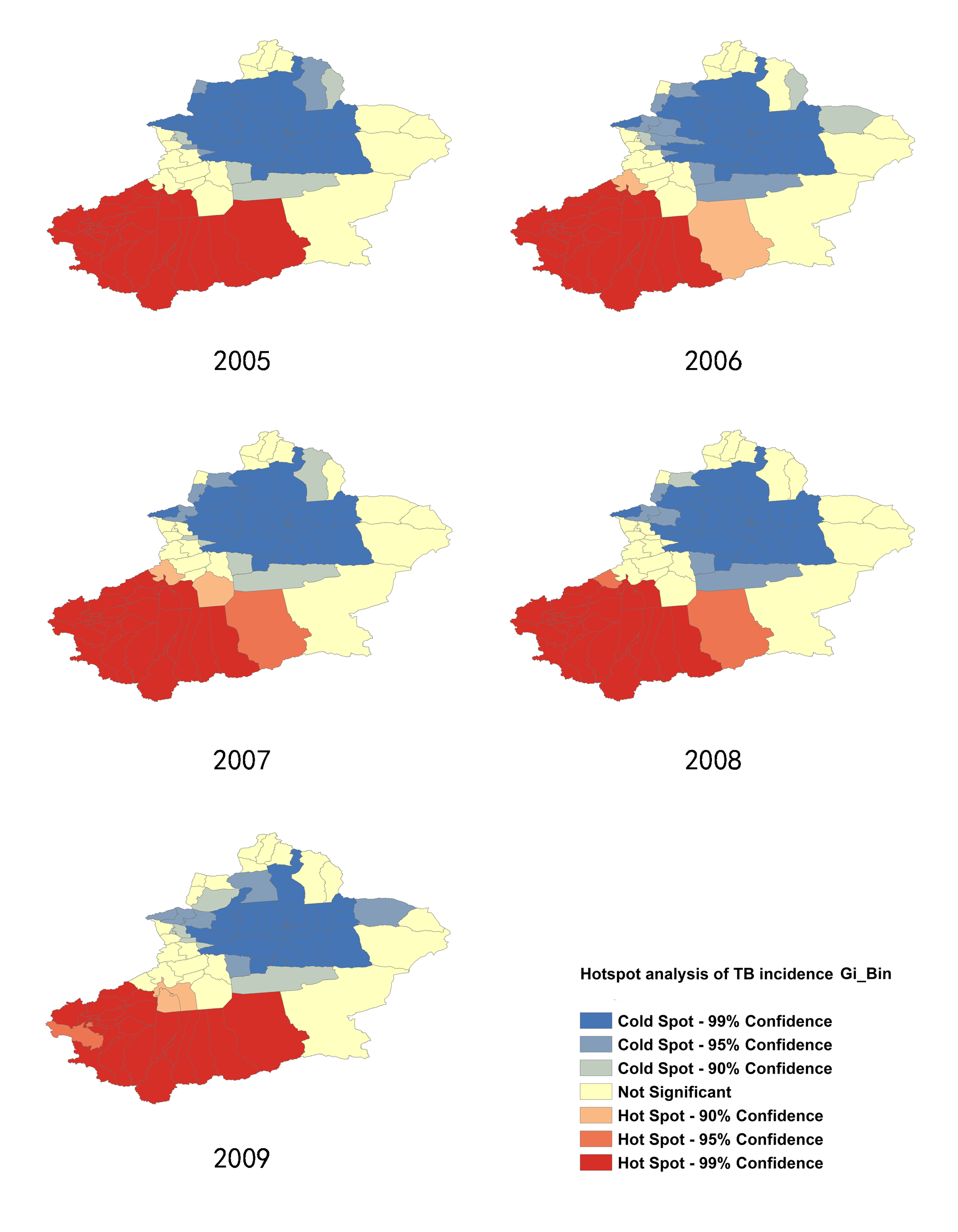

Supplement: S2 Fig — (TIF) [file pone.0144010.s002.TIF]

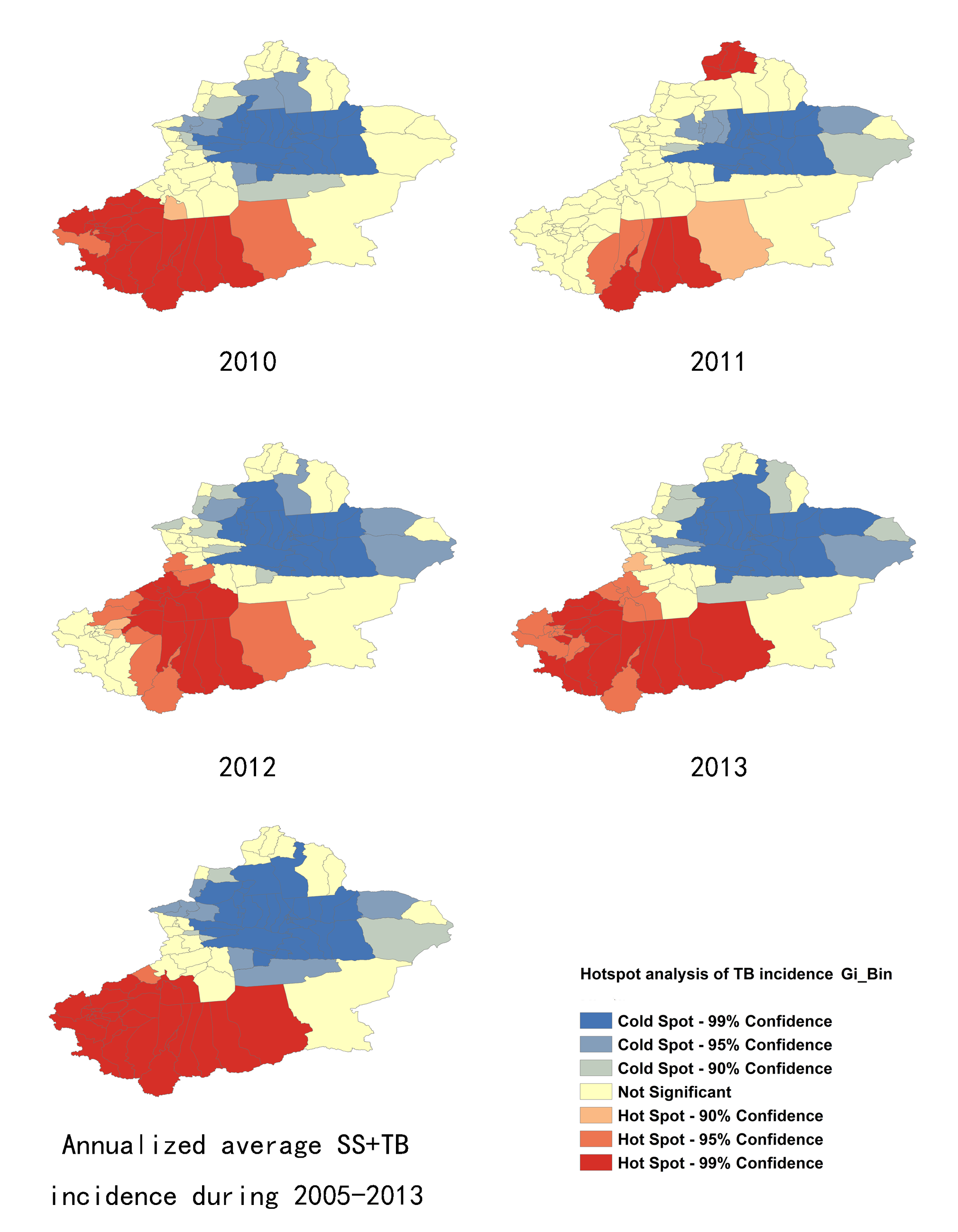

Supplement: S3 Fig — (TIF) [file pone.0144010.s003.TIF]

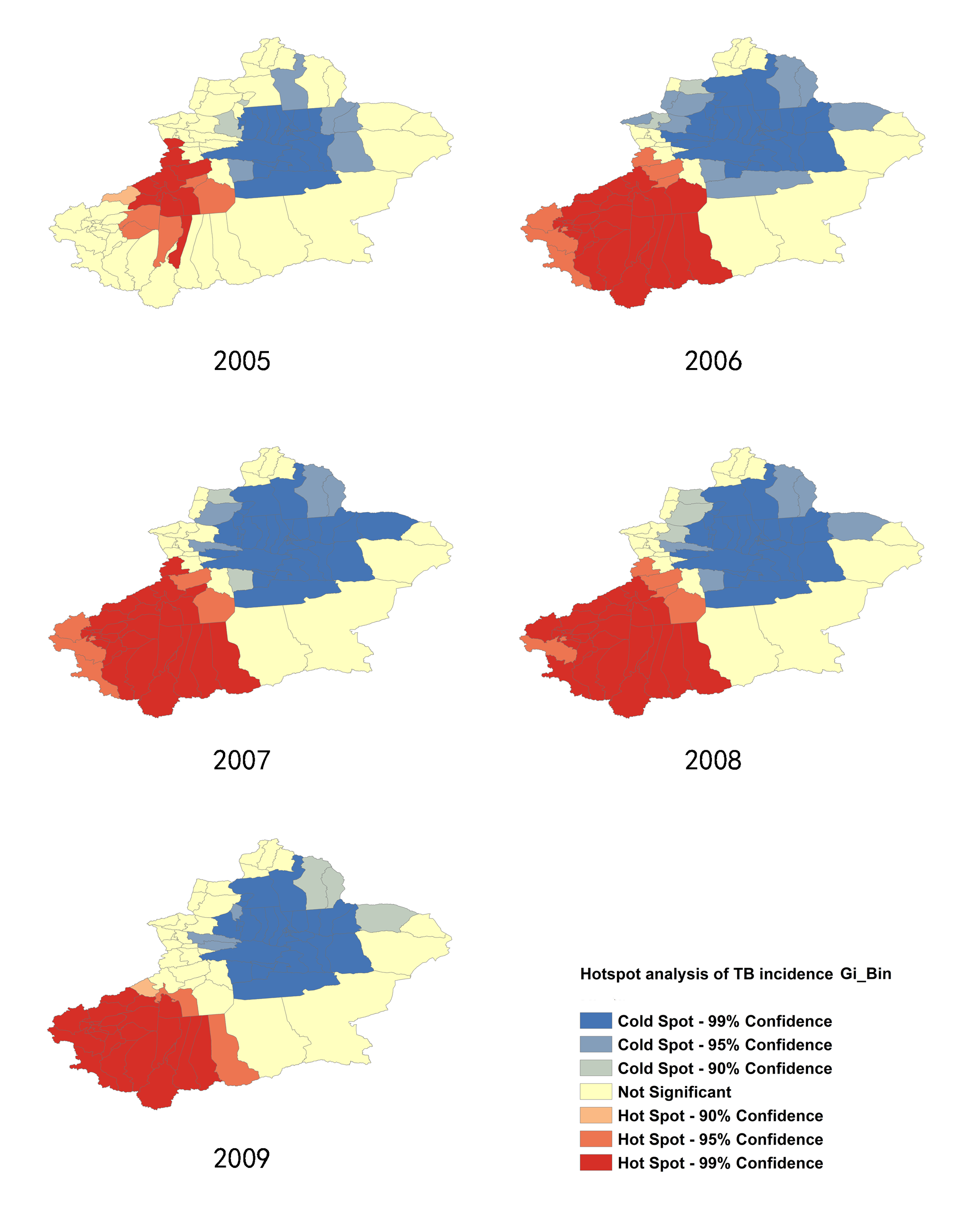

Supplement: S4 Fig — (TIF) [file pone.0144010.s004.TIF]

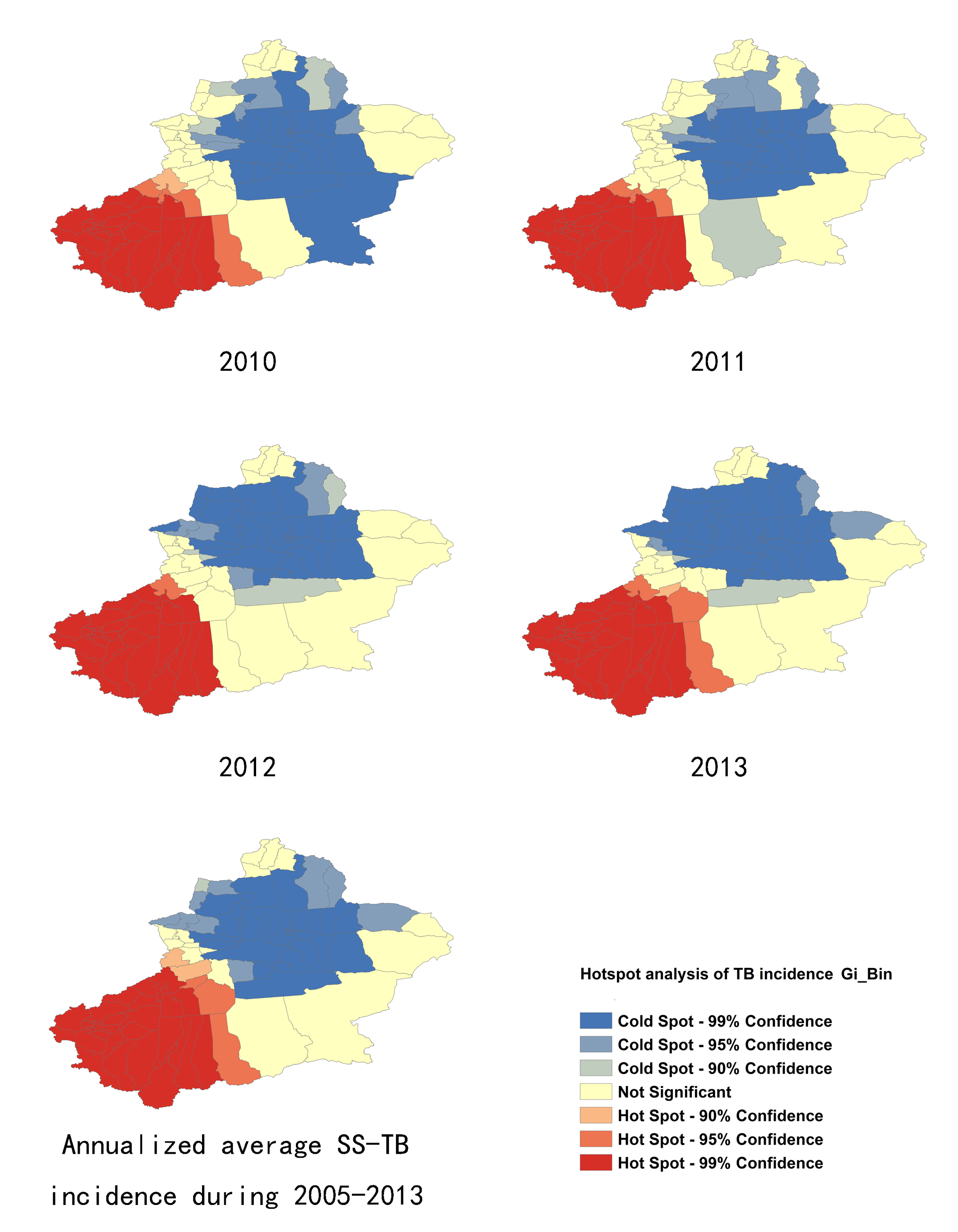

Supplement: S5 Fig — (TIF) [file pone.0144010.s005.TIF]
